# Aluminium phthalocyanine-mediated photodynamic therapy induces ATM-related DNA damage response and apoptosis in human oesophageal cancer cells

**DOI:** 10.3389/fonc.2024.1338802

**Published:** 2024-01-29

**Authors:** Onyisi Christiana Didamson, Rahul Chandran, Heidi Abrahamse

**Affiliations:** Laser Research Centre, Faculty of Health Sciences, University of Johannesburg, Johannesburg, South Africa

**Keywords:** photodynamic therapy, oxidative stress, cell cycle, DNA damage response, cell death, apoptosis

## Abstract

**Introduction:**

Photodynamic therapy (PDT) is a light-based technique used in the treatment of malignant and non-malignant tissue. Aluminium-phthalocyanine chloride tetra sulfonate (AlPcS4Cl)-mediated PDT has been well investigated on several cancer types, including oesophageal cancer. However, the effects of (AlPcS4Cl)-mediated PDT on DNA damage response and the mechanism of cell death in oesophageal cancer needs further investigation.

**Methods:**

Here, we examined the in vitro effects of AlPcS_4_Cl-mediated PDT on cell cycle, DNA damage response, oxidative stress, and intrinsic apoptotic cell death pathway in HKESC-1 oesophageal cancer cells. The HKESC-1 cells were exposed to PDT using a semiconductor laser diode (673.2 nm, 5 J/cm^2^ fluency). Cell viability and cytotoxicity were determined by the ATP cell viability assay and the lactate dehydrogenase (LDH) release assay, respectively. Cell cycle and DNA damage response (DDR) analyses were conducted using the Muse™ cell cycle kit and the Muse^®^ multi-color DNA damage kit, respectively. The mode of cell death was identified using the Annexin V-FITC/PI detection assay and Muse® Autophagy LC3 antibody-based kit. The intrinsic apoptotic pathway was investigated by measuring the cellular reactive oxygen species (ROS) levels, mitochondrial membrane potential (ΔΨm) function, cytochrome c levels and the activity of caspase 3/7 enzymes.

**Results:**

The results show that AlPcS_4_Cl-based PDT reduced cell viability, induced cytotoxicity, cell cycle arrest at the G0/G1 phase, and DNA double-strand break (DSB) through the upregulation of the ataxia telangiectasia mutated (ATM), a DNA damage sensor. In addition, the findings showed that AlPcS_4_Cl-based PDT induced cell death via apoptosis, which is observed through increased ROS production, reduced ΔΨm, increased cytochrome c release, and activation of caspase 3/7 enzyme. Finally, no autophagy was observed in the AlPcS_4_Cl-mediated PDT-treated cells.

**Conclusion:**

Our findings showed that apoptotic cell death is the main cell death mechanism triggered by AlPcS_4_Cl-mediated PDT in oesophageal cancer cells.

## 1 Introduction

Oesophageal cancer, a tumor arising from the oesophagus, poses a serious global health challenge with increased cancer-related illness and death rates. Global Cancer Observatory observed that the incidence-to-death rate ratio of oesophageal cancer is increasingly alarming. Oesophageal cancer is associated with delayed diagnosis due to lack of symptoms, resulting in inadequate treatment outcomes and reduced survival rates (1, 2). Conventional treatment approaches, which consist of surgery, chemotherapy, and radiotherapy, have made significant advancements in the diagnosis and treatment of oesophageal cancer, notwithstanding the inability to eliminate this tumor is still a great source of concern. Hence, there is an urgent need for other therapeutic options that can preferentially attack tumor cells while sparing healthy tissues. In recent years, photodynamic therapy (PDT) has evolved as a potential treatment strategy for different types of cancers, including oesophageal cancer (3–7). PDT is a less invasive light therapy that utilizes the cytotoxic actions of a photoactivated agent known as photosensitizer (PS) (8). PDT has the potential to eliminate tumor cells by triggering programmed cell death (apoptosis and autophagy) and non-programmed cell death (necrosis) (9, 10). Clinically, PDT has been approved for the treatment of benign, superficial and late-stage oesophageal cancers either as curative or salvage therapy (5–7, 11). In clinical practice, PDT is performed via a flexible laser endoscopy system. The detailed procedures on how the PS is delivered and how the tumor of interest is differentiated from normal tissue can be found in references (11–15).

Various PSs have been employed for oesophageal cancer PDT, including porfimer sodium, porphyrins, chlorins, pheophorbides, 5-aminolevulinic acid and phthalocyanine (16). Phthalocyanine and its subtypes have been widely evaluated for the therapeutic application of oesophageal cancer (17–19). This is due to its high absorption wavelength in the therapeutic window and its excellent induction of reactive oxygen species (ROS), which is crucial in promoting cell damage. Aluminium-phthalocyanine chloride tetra sulfonate (AlPcS_4_Cl), a sub-class of phthalocyanine, has been extensively employed for PDT application on several tumor types, including oesophageal cancer (3, 20–23). It has been established that the subcellular localization of any PSs decides the effectiveness of PDT and, consequently, the cell death pathway (24). Studies have demonstrated that AlPcS_4_Cl is localized in the mitochondria, lysosome, cytoplasm, and nucleus (20, 25). Suggesting that AlPcS_4_Cl localization in these organelles dictates the mode of cell death. Reports show that PSs accumulating in the mitochondria, lysosomes, and nuclei promote apoptosis, while cytoplasm/plasma membrane localization causes necrosis. Aside from PS localization, the PS concentration and the light dose also influence the mode of cell death. In addition, PSs localizing in the nuclei induce apoptosis through a DNA damage-dependent pathway (26–29).

Studies have shown that DNA-damaging drugs could trigger the DNA damage response (DDR) pathways (30–32). The DDR pathway is usually confirmed by increased expression of specific proteins, which include ataxia telangiectasia mutated (ATM). ATM is a key protein that is upregulated by DNA double-strand breaks (DDSB). It functions as an upstream protein that facilitates the phosphorylation of histone H2A variant X (H2A.X) and the activation of p53 for various types of DNA repair responses (33, 34). Previous reports have shown that phthalocyanine-based photosensitizers can induce DNA damage and cell death across multiple cancer types (35, 36). However, the mechanism by which AlPcS_4_Cl induce DNA damage response and cell death in the oesophageal cancer cell is not clear. In this work, we evaluated the impacts of AlPcS_4_Cl-mediated PDT in triggering oxidative stress, DNA damage response, cell cycle arrest and apoptosis via the intrinsic pathway in oesophageal cancer cells. The results showed that AlPcS_4_Cl-mediated PDT induced oxidative imbalance, G0/G1 phase cell cycle arrest, caused DNA damage via DNA double-strand break and the upregulation of ATM kinase. In addition, AlPcS_4_Cl-mediated PDT triggered cell death via the intrinsic mitochondrial apoptotic pathway *in vitro* in human oesophageal cancer cells.

## 2 Materials and methods

### 2.1 Cell culture

The human oesophageal cancer cell line (HKESC-1 (RRID: CVCL_D568)) was commercially obtained from Cellonex, Johannesburg, South Africa. The HKESC-1 was cultivated in Dulbecco’s modified Eagle’s medium (DMEM) (D5796, Sigma-Aldrich) with the addition of 10% Foetal Bovine Serum (FBS) (Biochrom, S0615), 1 mM sodium pyruvate, 1% antibiotic (Amphotericin B and Penicillin-streptomycin). The cells were sustained and kept in a tissue culture incubator with the following conditions: 5% CO_2_, 80% humidity and 37°C temperature.

### 2.2 Preparation of AlPcS_4_Cl photosensitizer stock solution and UV-Vis spectrophotometry analysis

The AlPcS_4_Cl (AlPcS-834, Frontier Scientific) with a chemical formula C_32_H_16_AlClN_8_O_12_S and a molecular weight of 895.21 g/mol was protected from light and stored at room temperature. A stock concentration of 0.001 M was prepared by dissolving 0.013428 g of AlPCS_4_Cl in 15 mL of deionized water. The prepared stock solution was aliquoted, wrapped with aluminium foil, protected from light and stored in the fridge at 4°C. The UV-Vis spectrophotometry analysis was performed to determine the absorption spectrum of AlPcS_4_Cl, and the wavelength required for PDT application. The GENOVA NANO spectrophotometer (Jenway, 67912) was used to measure the absorption spectrum of AlPcS_4_Cl solution through a wavelength range of 400-800 nm.

### 2.3 Intracellular localization of AlPcS_4_Cl

The cellular localization of AlPcS_4_Cl in HKESC-1 oesophageal cancer cells was conducted as previously described (20). Cells were grown at a seeding density of 5 x 10^5^ cells on glass coverslips in 35 mm culture dishes containing 2 mL of complete pre-warmed DMEM culture medium. The cells were maintained in 5% CO_2_, 85% humidity, and a 37°C incubator until they were attached. After attachment, the cells were washed three times with HBSS (Hanks’ Balanced Salt Solution) and then replaced with 2 mL of fresh pre-warmed complete DMEM culture media containing 20 µM of AlPcS_4_Cl. The cells were incubated for four hours to allow for effective intracellular accumulation. After incubation, the cells were rinsed three times with HBSS and fixed with 1 mL of paraformaldehyde (4%) for 15 minutes. The cells were rinsed, and 1 mL of 0.5% Triton X-100 was applied and allowed to stay for 10 minutes for permeabilization. The cells were rinsed again, and the cells’ organelles, such as mitochondria, lysosomes, and endoplasmic reticulum (ER), were labeled with 50µL of Mito-Tracker (100 nM) (M7514, Invitrogen™)), Lyso-Tracker (65 nM) (L7526, Invitrogen™), and ER-Tracker (65 nM) (E12353, Invitrogen™)), accordingly. The cells were kept on ice for 30 minutes and protected from light. Afterwards, the cells were rinsed, stained with 200 µL of 40,6- diamidino-2-phenylindole (DAPI) (D1306, Invitrogen™) and incubated for 5 minutes at room temperature. The coverslips were washed and mounted onto glass slides using a mounting media. Images were viewed using a Carl Zeiss Axio Z1 microscope with a 20x objective using the following filters: FITC (for Mito-tracker, lysotracker and ER-tracker), DAPI (nucleus), and AF660 filters for AlPcS_4_Cl. Zeiss Zen Blue 3.7 software was used for image acquisition.

### 2.4 Dose-response, laser parameters and PDT treatment

HKESC-1 oesophageal cancer cells were cultured at a seeding density of 5 x 10^5^ cells in 35 mm culture plates containing 2 mL of complete DMEM culture medium. The cells were placed in a 5% CO2, 85% humidity incubator, and 37°C incubator for 24 hours for adequate attachment. After 24 hours of incubation, the cells were rinsed thrice with HBSS and then replaced with 2 mL of fresh pre-warmed complete DMEM culture media containing 1.25, 2.5, 5, 10, and 20 µM of AlPcS_4_Cl. The cells were incubated for four hours to allow for effective intracellular accumulation, as previously reported (20, 37). The cells were grouped into two: AlPcS_4_Cl at 0 J/cm^2^ (no PDT) and AlPcS_4_Cl-PDT at 5 J/cm^2^. After incubation, the AlPcS_4_Cl-PDT cells were rinsed three times with HBSS, and 1 mL of 1 x phosphate-buffered saline (PBS) was added to the plates and then ready for irradiation. A 673.2 nm semiconductor laser diode (Arroyo, High Power Laser, National Laser Centre of South Africa) with a power output of 97 mW was utilized for irradiation. Prior to irradiation, the power output was measured with the field mate laser power meter and the reading obtained was used to determine the irradiating time. The cells were irradiated for eight minutes at a fluency of 5 J/cm^2^. After irradiation, the 1 x PBS was removed, and the cells were replenished with 2 mL of a complete DMEM culture medium. The cells with no PDT AlPcS_4_Cl at 0 J/cm^2^ were rinsed three times with HBSS and replaced with 2 mL of a complete DMEM culture medium. The two groups of cells were further incubated for 24 hours in a 5% CO_2_ and 85% humified incubator at a temperature of 37°C. All irradiation procedures were conducted in the dark to prevent background light interaction. The formula below was used to estimate the fluency.


$$Fluency\;\left(J/cm^{2}\right)\;=\;time\;\left(s\right)\;\left[power\left(W\right)/surface\;\left(cm^{2}\right)\right].$$

P cell viability and cytotoxicity assays were conducted to determine the effects of AlPcS_4_Cl on HKESC-1 cells with various concentrations (1.25, 2.5, 5, 10, and 20 µM) with or without PDT treatment. A dose response was performed on HKESC-1 cells to obtain the appropriate concentration of AlPcS_4_Cl required for the downstream application of PDT on HKESC-1 cells. The ATP cell viability results were used to identify the 50% inhibitory concentration (IC_50_). The IC_50_ concentration obtained from the cellular dose response of AlPcS_4_Cl-mediated PDT on oesophageal cancer cells was used to assess the mode of cell death triggered by AlPcS_4_Cl in Section 2.7.

### 2.5 Adenosine triphosphate cell viability assay

Adenosine triphosphate (ATP) cell viability was conducted using the CellTiter-Glo^®^ 3D luminescence (G968, Promega). Briefly, 50 μL of cell suspension and 50 μL of CellTiter-Glo^®^ 3D reagent were added to a 96-well plate and mixed properly, then incubated at room temperature for 10 minutes in the dark. The PerkinElmer VICTOR Nivo™ plate reader was used to measure the ATP luminescence.

### 2.6 Lactate dehydrogenase release assay

The cell membrane functionality of HKESC-1 cells was examined by measuring the efflux of lactate dehydrogenase (LDH) enzyme from impaired cell membranes of HKESC-1 cells. To evaluate LDH cytotoxicity, a CytoTox 96^®^ Non-Radioactive Cytotoxicity assay (G1780, Promega) was used. Briefly, 50 μL of reconstituted reagent and equal volume cell culture medium were added into a 96-well plate and incubated for 30 minutes at room temperature. Absorbance was measured at 490 nm using the PerkinElmer VICTOR Nivo™ plate reader.

### 2.7 Mode of cell death evaluation

The mode of cell death induced by AlPcS_4_Cl-mediated PDT was evaluated using the IC_50_ concentration of 5 μM obtained from the cellular dose response in Section 2.4. HKESC-1 cells (5.0 x 10^5^) were grown in 35 mm culture plates, and PDT treatment was performed as highlighted in Section 2.4. The cells were grouped into four categories: control, laser-only, AlPcS_4_Cl and AlPcS_4_Cl-PDT.

#### 2.7.1 Cell morphology evaluation

Twenty-four hours following PDT, cells were checked for cellular morphological variation using an inverted light microscope (Olympus CKX41Q4) attached to a camera. The various oesophageal cancer cell groups were examined, and images were obtained using the 20X objectives with 100 µm scale bars. The images were checked for morphological variation in cell size, shape, structure, and attachment to the culture plate and compared in relation to the control cells.

#### 2.7.2 Cell cycle assessment

Cell cycle assessment was carried out with the Muse™ Cell Cycle Kit (MCH100106, Millipore) using the assay’s protocol guide. After 24-hour PDT exposure, the cells were harvested, 1.0 x 10^6^ cells/mL were washed 1 x PBS, and the supernatant was expelled. The cells were fixed in a newly prepared ice-cold 70% ethanol for 3 hours in a -20°C freezer. Following fixation, 200 μL of the cells were transferred into a new tube. The cells were washed in 1 x PBS, and the supernatant was discarded. The cells were resuspended in the Muse^®^ Cell Cycle reagent (200 μL), kept at ambient temperature for 30 minutes without light, and analyzed using the Guava Muse Cell Analyzer (Millipore).

#### 2.7.3 DNA damage response assessment

DNA damage response assessment was conducted with the Muse^®^ Multi-Color DNA Damage Kit (MCH200107, Luminex) following the kit’s instructions. Following 24-hour post-PDT treatment, 50 μL volume of 1x assay buffer containing 1 x 10^5^ cells and 50 μL fixation buffer were dispensed into a 1.5 microtube, mixed, and incubated for 10 minutes at 2-4°C. The cells were washed, the supernatant eliminated and permeabilized with ice-cold 1x permeabilization buffer (100 μL) for 10 minutes at 2-4°C. The cells were stained with 100 μL of diluted antibody cocktail (containing anti-phospho-ATM (Ser1981)-PE and anti-phospho-Histone H2A.X (Ser139)-PECy5 antibodies) and incubated for 30 minutes at room temperature in the absence of light. The cells were washed, the supernatant expelled and resuspended in 1x assay buffer (200 μL). The cells were analyzed with the Muse^®^ Cell Analyzer.

#### 2.7.4 Annexin V-FITC/PI assay

To examine the cell death mechanism in the control and treatment group, we used the Annexin V-FITC/PI (556570, BD Pharmingen™) flow cytometry assay. The percentage of apoptotic and necrotic cells was measured following the manufacturer’s manual. In summary, after 24 hours post-PDT, cells were detached, rinsed in ice-chilled 1 x PBS, and then recovered in 500 μL of binding buffer. A 100 μL of cells were transferred into sterile 5 mL tubes. The cells were stained with an equal volume of 5 μL of annexin V-FITC and of propidium iodide (PI). The tubes were kept for 15 minutes at ambient temperature and covered with foil. Following the 15-minute incubation, 400 μL of 1X binding buffer was pipetted into the tubes and kept in the dark for 30 minutes. The cells were analyzed using the Becton Dickinson (BD) flow cytometer Accuri™ C6. Annexin V–FITC binding was measured using FL1filter (FITC), and PI was measured with FL2 filter [phycoerythrin (PE)]

#### 2.7.5 Detection of reactive oxygen species

Intracellular reactive oxygen species (ROS) levels were measured using the fluorescent probe 2′,7′-dichlorofluorescein diacetate (DCFDA/H2DCFD), cellular ROS assay (ab113851, Abcam). The HKESC-1 cells (10,000 cells/well) were grown in 96-well tissue culture plates for 24 hours for proper attachment. Twenty-four hours after PDT application, the cells were stained with 5 μM of DCFH-DA and kept for 10 minutes at 37°C temperature. Then the cells were added to a black plate of 96-well. The PerkinElmer VICTOR NivoTM plate reader was used to measure the fluorescence intensity using an excitation/emission filter of 485 nm/538 nm wavelength. Mitochondrial Membrane Potential Assessment

The rhodamine-123 (ab275545, Abcam) efflux flow cytometry assay, as previously described (21), was used to assess the mitochondrial membrane potential (ΔΨM). Rhodamine-123 is a fluorescent dye that localizes in mitochondria, and when the mitochondria membrane potential is impaired, the rhodamine leaks out, reducing the fluorescence intensity of the cell. Briefly, 24 hours following PDT treatments, cells were detached, harvested, and washed twice in 1x PBS. The cell pellets were recovered in 100 μL of 1 x PBS and treated in 25 μM of rhodamine 123. The cells were incubated in the dark at ambient temperature for 15 minutes and then were washed. The cell pellets were suspended in 400 μL of 1 x PBS, homogenized and kept in the dark at room temperature for half an hour. The rhodamine-123 fluorescent intensity was measured using an Accuri™ C6 BD flow cytometer with the FL1-H filter and evaluated with BD CSampler software.

#### 2.7.6 Cytochrome c release assessment

The human cytochrome c ELISA assay (BMS263, Invitrogen) was utilized to measure the amount of cytochrome c release in the cell lysate. The test was performed according to the manufacturer’s directives. Briefly, 24 hours post-PDT, 1.5 x 10^6^ cells were lysed with 1 mL lysis buffer for one hour at room temperature. The cells were centrifuged for 15 minutes at 200 x g, and 5 μL of the lysate was diluted in 245 μL of 1 x assay buffer. One hundred microlitres of the diluted lysate and 50 μL of biotin-tagged anti-human cytochrome c antibody were added into the microwell strips. The microwell strips were incubated for two hours at room temperature and thereafter rinsed with wish wash buffer. Streptavidin-HRP secondary antibody (100 μL) was dispensed into the microwells and incubated for 60 minutes at ambient temperature. The microwells were washed, tetramethyl-benzidine substrate (100 μL) was added, and after 10 minutes, 100 μL of stop solution was added. The cytochrome c levels were measured using the PerkinElmer VICTOR Nivo™ microplate reader set at 450 nm.

### 2.8 Measurement of Caspase 3/7 activity

The Caspase-Glo 3/7 luminescent assay kit (G8090, Promega) was used to detect the caspase 3/7 levels. The assay was conducted using the manufacturer’s instructions. Briefly, 24 hours post-PDT, 50 μL volume each of Caspase-Glo 3/7 luminescence reagent and the cell suspension was added in a 96 microwell plate, mixed, and incubated for about 30 minutes to 3 hours at room temperature in the dark. The Caspase-Glo 3/7 luminescence produced was measured with the plate reader PerkinElmer, VCTOR Nivo™.

### 2.9 Autophagy evaluation

Autophagy detection was conducted using the Muse^®^ Autophagy LC3 antibody-based kit (MCH200109, Luminex). Briefly, cells were cultured at a cell concentration of 4.0 x 10^4^ cells/well in a 96-well plate for 24 hours and subjected to PDT. Following 24 hours post-PDT, the cells were treated with 200 μL of autophagy reagent A and incubated for 4 hours at 37°C. The cells were harvested, spun down at 300 x g for 5 minutes, and the supernatant was discarded. The cells were stained with 100 μL of diluted Anti-LC3 Alexa Fluor™ 555 antibody and kept at 2 – 4°C for half an hour without light. The cells were centrifuged, and the supernatant was eliminated. The residual cells were washed with 1x assay buffer, centrifuged, and the supernatant expelled. The residual cells received 200 μL of 1x assay buffer and were adequately mixed and measured using the Muse^®^ Cell Analyzer. The autophagy induction ratio was obtained by calculating the ratio of the mean autophagy intensity of the test sample and the mean autophagy intensity of the control sample. Values ≥1 denote autophagy induction, and ≤1 depicts no induction.

### 2.10 Statistical analysis

All experimental results were collated from three replicates of two independent experiments and are demonstrated as the mean ± SEM (standard error of the mean). Statistics were conducted using a one-way variance analysis using the GraphPad Prism version 5 (GraphPad Inc., San Diego, CA, USA). Statistical significance was set at P<0.05.

## 3 Results

### 3.1 Absorption spectrum and chemical structure of AlPcS_4_Cl

**Figure 1:**
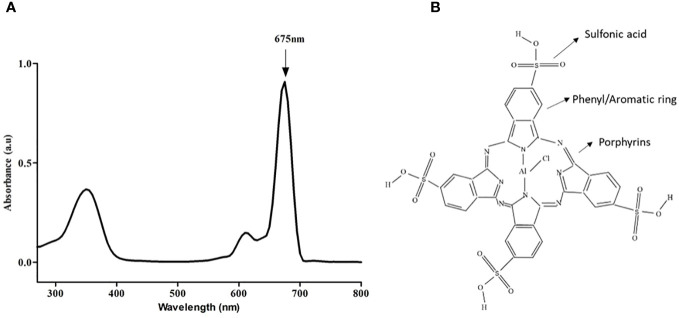
**(A)** Spectral absorption of AlPcS_4_Cl indicating the highest peak at 675 nm. **(B)** Chemical structure of AlPcS_4_Cl.

The absorption spectrum of AlPcS_4_Cl was analyzed using UV-Vis Spectrophotometry, and results showed the highest absorption at 675 nm when measured from 200 nm to 800 nm wavelength. **Figure 1** shows the wavelength at which AlPcS_4_Cl has the maximum absorption. Also present in this spectrum (**Figure 1A**) is a medium peak called the soret band at ± 350 nm. This wavelength can be used for fluorescence detection in photodiagnosis. For AlPcS_4_Cl-mediated PDT in this study, a 673.2 nm semiconductor diode laser was utilized. The structure of AlPcS_4_Cl shows the core porphyrin structure and the presence of the phenyl/aromatic rings and sulfonic acid functional groups (**Figure 1B**).

### 3.2 Intracellular localization of AlPcS_4_Cl

**Figure 2:**
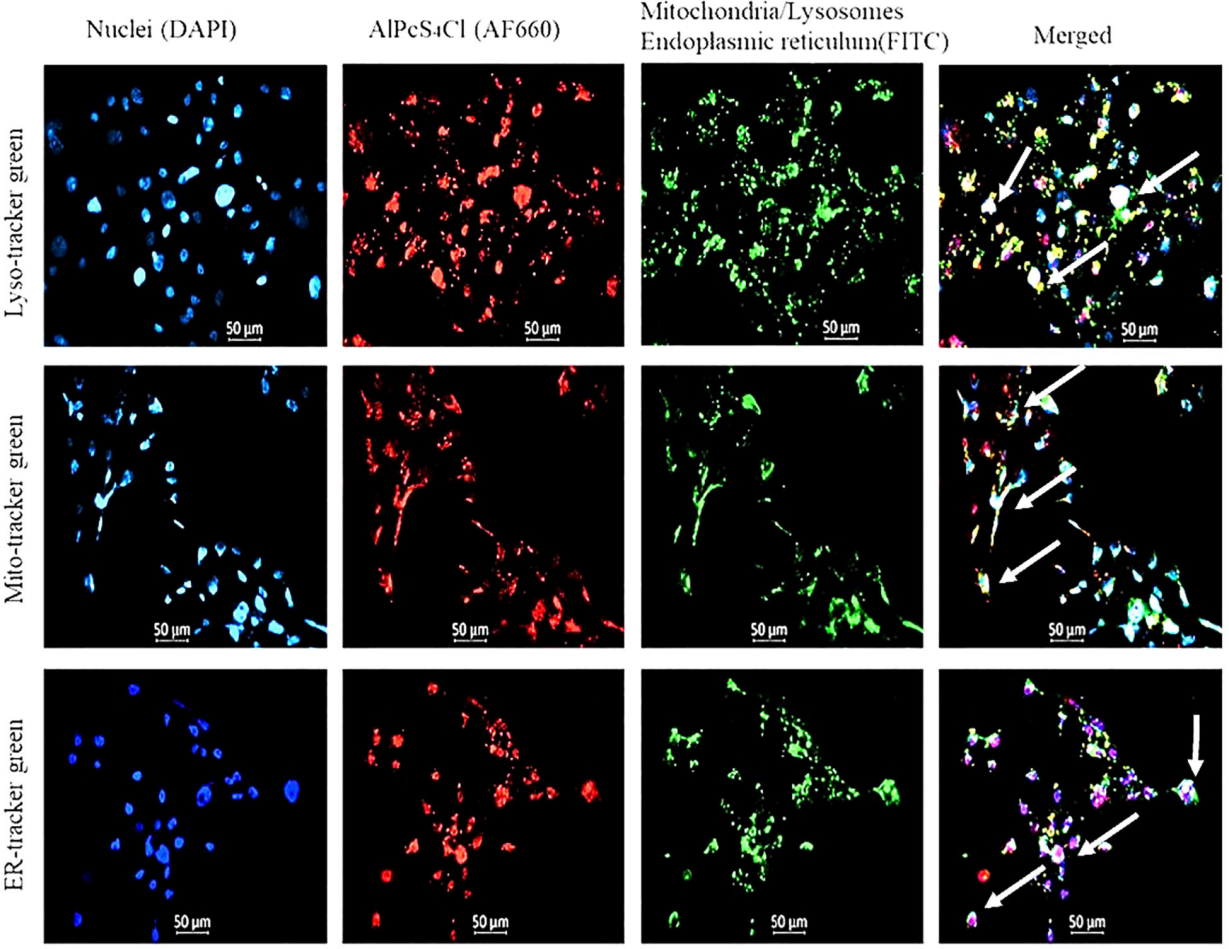
The nuclei are counterstained with DAPI (blue), mitochondria, lysosome, ER stained with FITC (green), and AlPcS_4_Cl auto fluoresces (red). The merged yellow/white and pink colours showed an accumulation of AlPcS_4_Cl in the mitochondria, lysosome, ER, and nucleus.

Intracellular localization of AlPcS_4_Cl in HKESC-1 oesophageal cancer cells was performed four hours post PS incubation using organelle-specific trackers labeled with fluorescence probe and observed with fluorescence microscopy. The following intracellular organelles, such as mitochondria, lysosome, and ER, were stained using the mito-tracker, lysotracker, and ER tracker fluorochrome, respectively. At the same time, the nuclei were counterstained with DAPI. In **Figure 2**, the blue fluorescence indicates the nuclei, the red fluorescence denotes AlPcS_4_Cl, and the green fluorescence represents the mitochondria, lysosomes, and ER. The merged yellow/white and pink colours showed an accumulation of AlPcS4Cl in the mitochondria, lysosome, ER, and nucleus.

### 3.3 Cellular viability and determination of 50% inhibitory concentration (IC_50_) of AlPcS_4_Cl in oesophageal cancer cells

**Figure 3:**
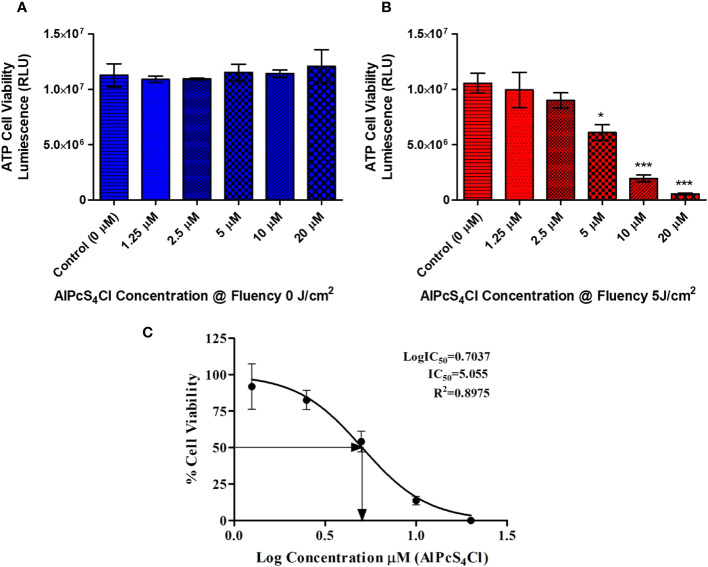
ATP cell viability of AlPcS_4_Cl treated oesophageal cancer cells using the ATP cell viability luminescence assay. The relative luminescent unit (RLU) is proportional to the ATP level in the cell/cell viability. **(A)** Control cells and cells treated with different amounts of AlPcS_4_Cl without photoactivation (0 J/cm^2^) displayed high ATP levels/viability. No statistically significant was observed. **(B)** Irradiated cells receiving varying concentrations of PDT at 5 J/cm^2^ exhibited reduced ATP levels and cell viability, with a significant reduction seen in the cells receiving 5 μM, 10 μM and 20 μM at 5 (*p<0.05, ***p< 0.001). **(C)** The IC_50_ viability concentration of AlPcS_4_Cl on HKESC-1 cells. The values displayed are mean ± SEM (standard error of the mean) of three replicate tests from two separate experiments.

An ATP cell viability analysis was performed to determine the ability of AlPcS_4_Cl to inhibit HKESC-1 cancer cells. High cell viability was seen in the control group, and the group administered AlPcS_4_Cl without irradiation (0 J/cm^2^) (**Figure 3A**). This result showed that AlPcS_4_Cl is non-toxic in the inactivated state. Conversely, the PDT-treated cells exposed to 5 J/cm^2^ irradiation showed a significant reduction of cellular ATP generation when compared to the control (*p<0.05, ***p<0.001) (**Figure 3B**), which is an indication of non-viable cells. This reduction follows a dose-dependent pattern: the higher the concentration, the lower the cell viability/ATP production. This observation demonstrated that AlPcS_4_Cl-mediated PDT inhibited cell viability and cell growth, consequently leading to cell death. Furthermore, we evaluated the IC_50_ concentration on the cancer cells to identify the appropriate concentration of AlPcS_4_Cl needed for downstream PDT application. The IC_50_ values were performed with ATP cell viability assay following 24 hours post-PDT using the following concentrations of AlPcS_4_Cl (1.25, 2.5, 5, 10, and 20 µM). The IC_50_ concentration was estimated by calculating the inhibition of luminescence of ATP cellular viability using the log(inhibitor) versus normalized response-variable slope equation in GraphPad Prism, version 5.0.

$Y=100/\left(1\;+\;10\hat{\;}\left(\left(LogIC50-X\right)*HillSlope\right))\right)$ ^1^

Where Y represent the % inhibition of cell viability and X the concentration of AlPcS_4_Cl.

In **Figure 3C**, the result showed that AlPcS_4_Cl exhibited an IC_50_ value of 5.05µM on oesophageal cancer cells. The IC_50_ of 5 µM was used for all cell death PDT investigations in Section 3.5.

### 3.4 Lactate dehydrogenase release mediated by AlPcS_4_Cl-PDT

**Figure 4:**
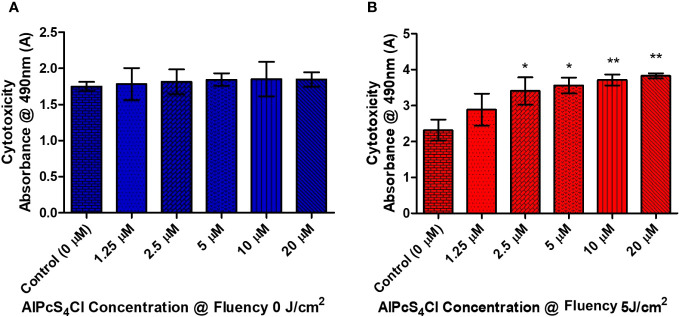
The cytotoxic effects of AlPcS_4_Cl on oesophageal cancer cells. The LDH release cytotoxicity assay on HKESC-1 cells was measured 24 hours following PDT. **(A)** The amount of LDH leakage in the control groups at 0 J/cm^2^. Demonstrating no significance. **(B)** the PDT-treated cells exhibited a high statistical significance difference in relation to the control (*p<.05, **p< 0.001). The results are depicted as the mean ± SEM of three repeats from two individual experiments.

To determine the cytotoxicity impact of AlPcS_4_Cl on HKESC-1 oesophageal cancer cells, a lactate dehydrogenase (LDH) release cytotoxicity assay was conducted. The LDH assay is established on the principle that cells with disrupted cell membranes expel LDH, denoting cytotoxicity. The cytotoxicity impact on the cancer cells was examined 24 hours after PDT by quantifying the amount of LDH enzyme that was leaked into the culture media. Low LDH activities were observed in the control cells and the cells exposed to AlPcS_4_Cl 0 J/cm^2^ (**Figure 4A**). This result further confirmed the non-toxic effect of AlPcS_4_Cl when not activated. **Figure 4B** showed that the cells treated with PDT showed a dose-responsive high release of LDH, indicating cell membrane impairment. The results showed that AlPcS_4_Cl-mediated PDT drives cell membrane disruption and cell death.

### 3.5 Evaluation of cell death mechanism

#### 3.5.1 Effects of AlPcS_4_Cl-mediated PDT on the morphology of oesophageal cancer

**Figure 5:**
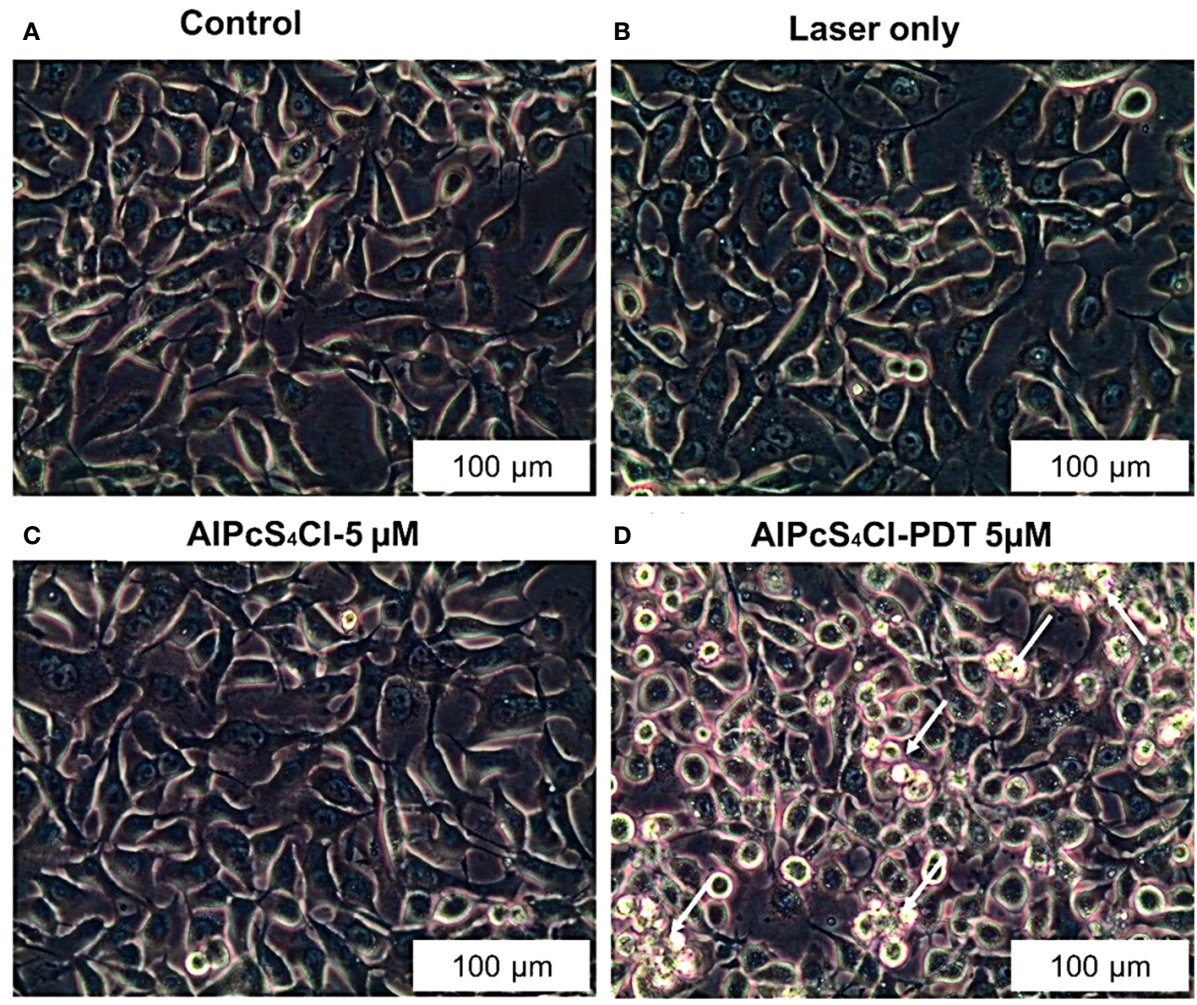
Morphological examination of oesophageal cancer cells as shown with 20X objective magnification displaying healthy cellular structure in the control, laser-only, AlPcS_4_Cl alone **(A–C)** and significant cellular impairment in theAlPcS_4_Cl-mediated PDT treated cells **(D)**.

The different forms of cell death mechanisms can be identified based on their cellular morphological changes. The morphological examination was carried out 24 hours after PDT on four groups of oesophageal cancer cells (control, laser-only, AlPcS_4_Cl and AlPcS_4_Cl-PDT) using an IC_50_ of 5µM. The control, laser-only and AlPcS_4_Cl displayed active proliferative cells with no morphological changes (**Figures 5A–C**). The AlPcS_4_Cl-PDT group irradiated at 5 J/cm^2^ showed significant morphological alterations (**Figure 5D**). The morphological changes noted were cell contraction, blebbing, cell rounding up, and damaged cell membrane. These features were marks of cell death events.

#### 3.5.2 AlPcS_4_Cl-mediated PDT induced cell cycle arrest at the G0/G1 phase in oesophageal cancer cells

**Figure 6:**
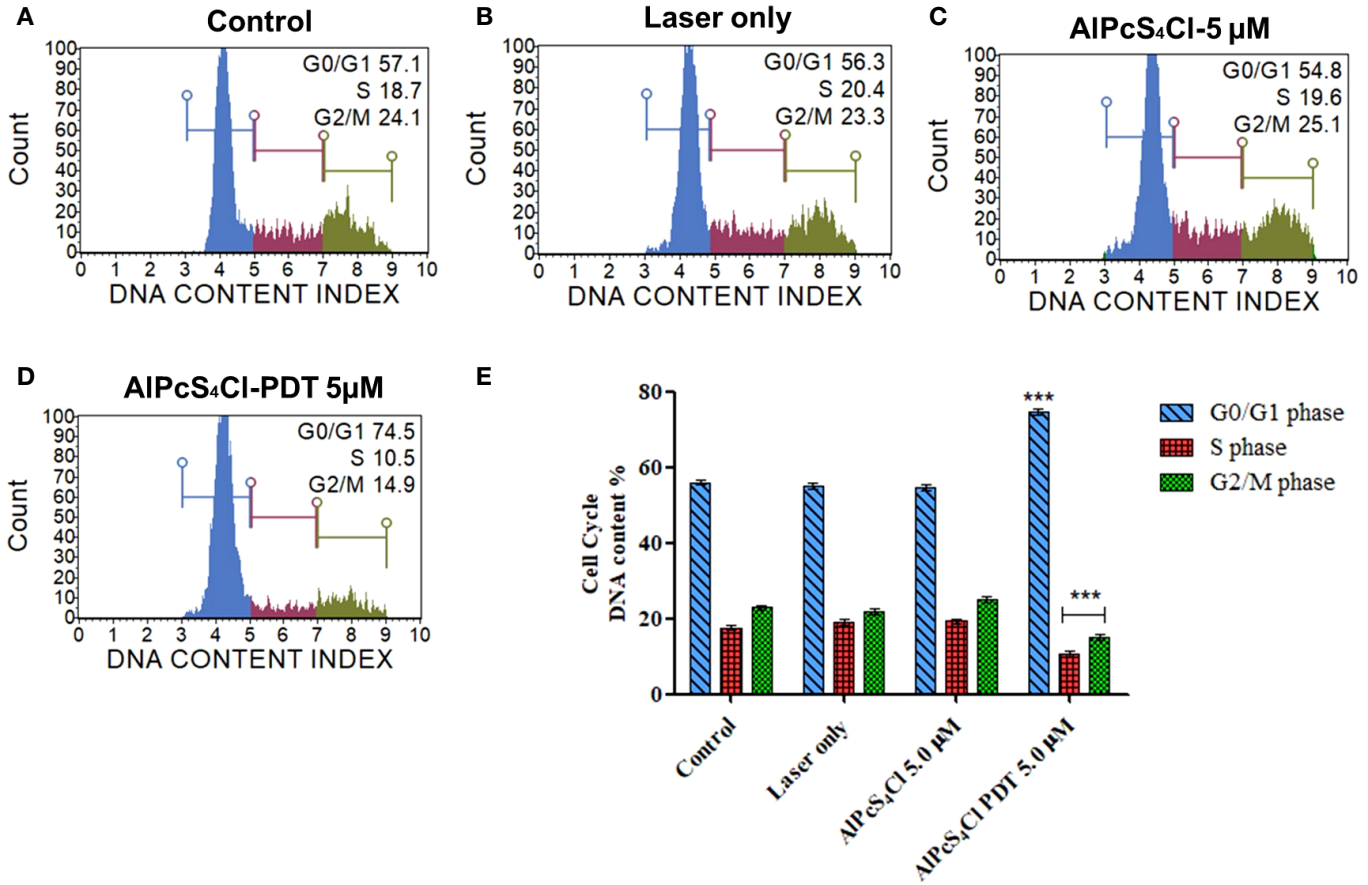
Cell cycle checkpoint evaluation of oesophageal cancer cells. The distribution of the respective cell cycle checkpoint phases **(A)** control untreated cells, **(B)** laser-only, **(C)** AlPcS_4_Cl and **(D)** AlPcS_4_Cl-PDT cells. The blue, red and green histograms represent the G0/G1, S and G2/M cell cycle checkpoint phases, respectively. **(E)** Percentage evaluation of three assays from two separate cell cycle analyses, with AlPcS_4_Cl-PDT showing G0/G1 cell cycle arrest compared to the control, laser and AlPcS_4_Cl only cells (***p< 0.001). The values shown are the mean ± SEM of triplicate tests from two independent experiments.

Aggressive cell growth is a major characteristic of tumor cells, which is usually driven by dysregulation of the cell cycle. The Muse Cell cycle kit was employed to investigate the effects of AlPcS_4_Cl-mediated PDT on the cell cycle. The cell cycle distribution results (**Figures 6A–D**) following 24-hour post-PDT exposure displayed the DNA content of the various cell cycle phases in the different experiment groups using Muse Cell Analyzer. Increased DNA content at the G0/G1 phase was observed in the AlPcS_4_Cl-PDT treated cells when compared with the control cells(***p<0.001) (**Figure 6E** blue bars). The laser-only and AlPcS_4_Cl-only groups displayed identical G0/G1 phase features with that of the control cells. The S phase (**Figure 6E** red bars) showed reduced DNA contents in the cells exposed to AlPcS_4_Cl-PDT treatment in relation to the control cells (***p<0.001). No significance was found in the laser-only and AlPcS_4_Cl-only groups relative to the control cells. The G2/M phase in **Figure 6E** (green bars) exhibited a significant reduction in the DNA contents in the cells receiving AlPcS_4_Cl-PDT treatment (***p<0.001), and no significance was demonstrated in the laser-only and AlPcS_4_Cl cells. These findings indicate that AlPcS_4_Cl-PDT treatment induces cell cycle arrest at the G0/G1 phase in oesophageal cancer cells. In addition, a significant defect in the G2/M checkpoint activity was noted in the AlPcS_4_Cl-PDT treated cells.

#### 3.5.3 DNA damage response induced by AlPcS_4_Cl-PDT

**Figure 7:**
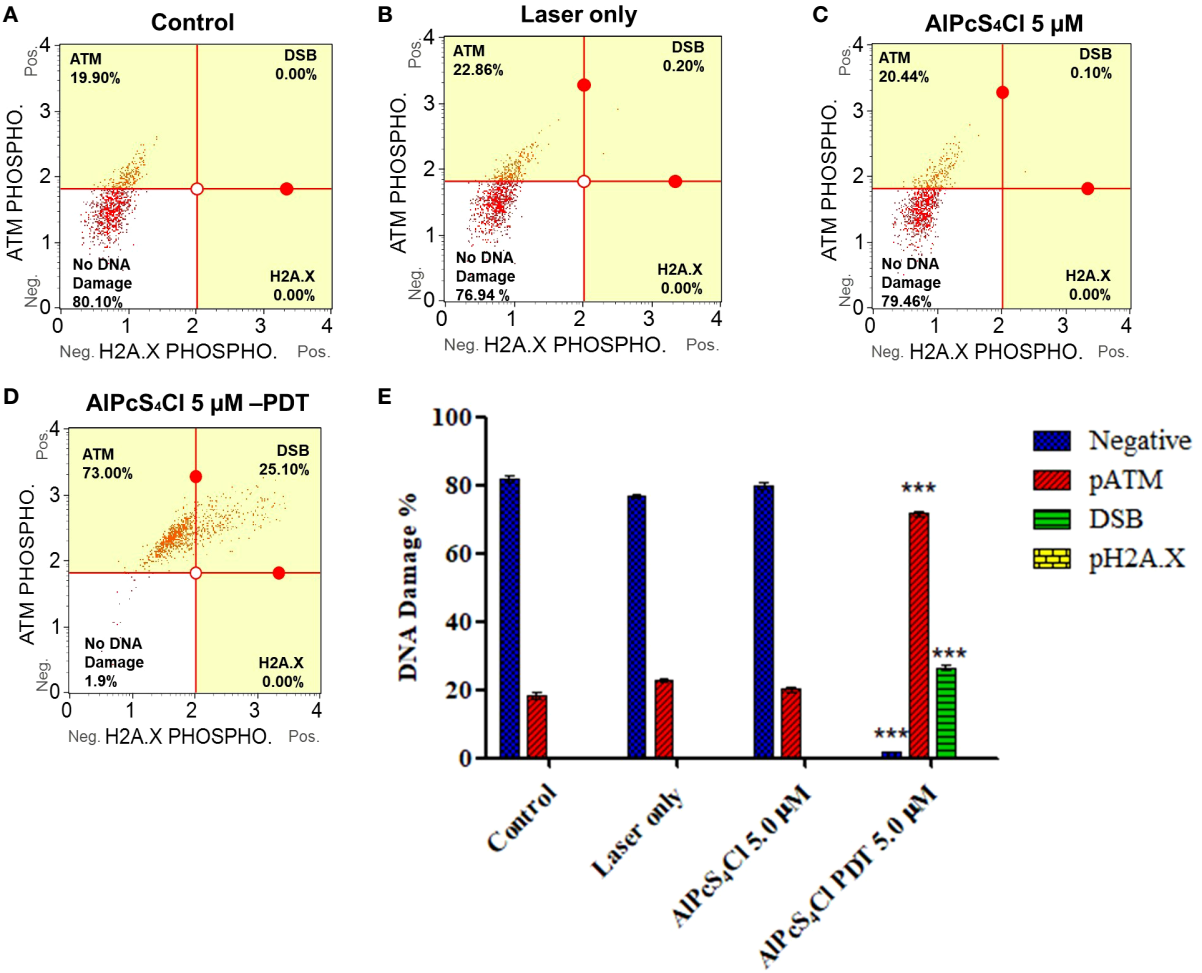
Quadrant profiles of DNA damage response assessment of oesophageal cancer cells exposure to treatment. Upper left: phosphorylated ATM, lower left: no DNA damage, upper right: double-strand break (DSB) (co-expressing ATM, H2A.X) and lower right: phosphorylated H2A.X. **(A)** control untreated cells, **(B)** laser-only, **(C)** AlPcS_4_Cl and **(D)** AlPcS_4_Cl-PDT. **(E)** DNA damage response of two independent triplicate tests depicted in bar graphs. The pATM-activated cell (red bars) showed increased activity in AlPcS_4_Cl-PDT, treated oesophageal cancer cells compared to the control (***p<0.001). The double-strand break (DSB) (green bars, co-expressing pATM/pH2A.X) showed a significant increase in the induction of DSB in the cells exposed to AlPcS_4_Cl-PDT relative to the control cells (***p<0.001). No significant expression of pH2A.X was observed in all the groups. The no DNA damage (blue bars) shows a significant reduction in the AlPcS_4_Cl-PDT treated cells compared to the control (***p<0.001). The results are the mean ± SEM of triplicate assays from two independent experiments.

The Muse Multi-Color DNA Damage Kit was employed to investigate the DNA damage response proteins ATM (ataxia-telangiectasia mutated) and H2A.X (histone H2A variant X)using Muse Cell Analyzer. This assay concurrently detects the expression of phosphorylated ATM, H2A.X and detection of DNA double-strand break (DSB) (co-expression of ATM/H2A.X cells) in the control and AlPcS_4_Cl -PDT treated oesophageal cancer cells. **Figures 7A–D** displayed the DNA damage response distribution of oesophageal cancer cells in the various groups.

The exposure of oesophageal cancer cells to AlPcS_4_Cl-PDT in **Figure 7E** significantly triggered DNA damage, as demonstrated in the high expression of ATM phosphorylation and increased DNA DSBs relative to the control cells (***p<0.001) (**Figure 7E**, red and green bars). Nevertheless, no significant DNA damage was demonstrated in the laser-only and AlPcS_4_Cl cells. The findings showed that AlPcS_4_Cl-PDT, treated oesophageal cancer cells, triggered DNA damage response signaling via DNA double-strand break and ATM pathways.

#### 3.5.4 Induction of apoptosis by AlPcS_4_Cl-mediated PDT

**Figure 8:**
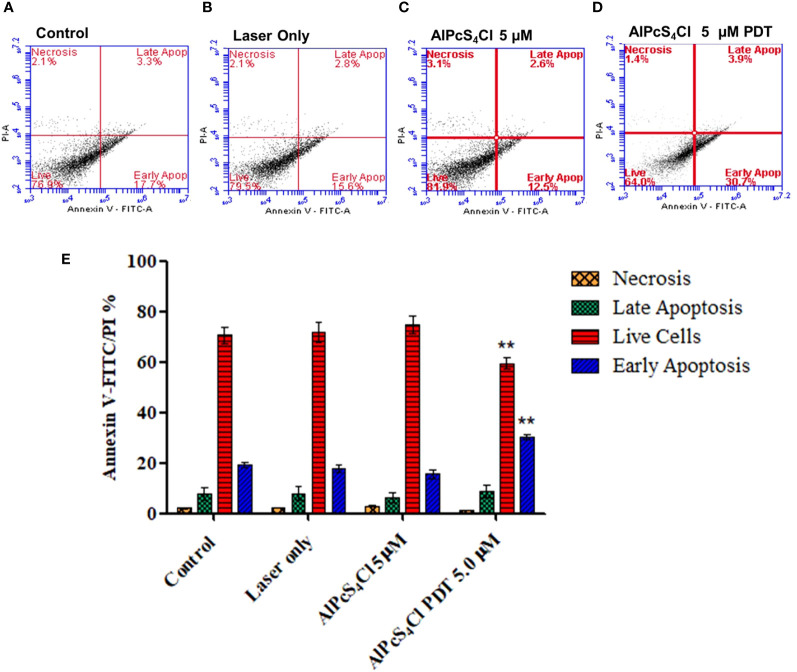
Cell death annexin V-FITC/PI of oesophageal cancer cells. Quadrant display of different phases of cell death **(A–D)** (control dark, control + irradiation, AlPcS_4_Cl dark and AlPcS_4_Cl-PDT). **(E)** Percentage quantification of triplicate tests from two independent cell death annexin V-FITC/PI assays on oesophageal cancer cells. The AlPcS_4_Cl-PDT treated cells showed a significant reduction of live cells (red bars) and a high percentage of early apoptosis (blue bars) compared to the control, laser only, and AlPcS_4_Cl cells (**p<0.01). The results are shown as the mean ± SEM of triplicate tests from two separate experiments.

Cell death evaluation was conducted with the annexin V-FITC/PI detection kit. In **Figures 8A–D**, the result showed a quadrant display of different phases of cell death (control, laser only, AlPcS_4_Cl and AlPcS_4_Cl-PDT). Oesophageal cancer cells treated with 5µM AlPcS_4_Cl-PDT showed a high percentage of early apoptotic cell death (30.7%) as against 17.7%, 15.6% and 12.5% in the control, laser-only and AlPcS_4_Cl-only cells respectively. The statistical significance of three repeated duplicate experiments was demonstrated in a bar graph, as seen in **Figure 8E**. The cells treated with AlPcS_4_Cl-PDT at 5 J/cm^2^ showed a significant reduction in viable live cells and increased early apoptosis (**p<0.01). However, no significant difference was seen among the treated cells and the various control cells in the necrotic and late apoptotic cell death fractions.

#### 3.5.5 AlPcS_4_Cl-mediated PDT induces ROS production and alters mitochondrial membrane potential

**Figure 9:**
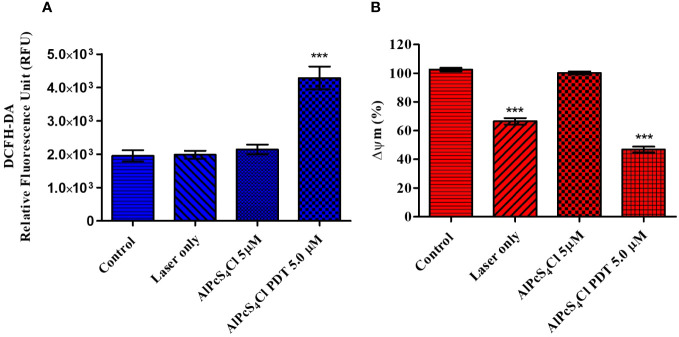
**(A)** The Cellular Reactive Oxygen Species (ROS) generation of AlPcS_4_Cl in HKESC-1 oesophageal cancer cells. Results showed an increased production of ROS in the AlPcS_4_Cl -PDT treated cells (***p< 0.001). **(B)** The impact of AlPcS_4_Cl on the Mitochondria membrane potential (Δψm) of oesophageal cancer cells. Rhodamine-123 efflux assay was used for evaluating the Δψm. Increased distortion of the Δψm was observed in control irradiated cells and AlPcS_4_Cl-PDT cells treated with 5 J/cm^2^ irradiation ***p< 0.001), with no significance shown in control dark and AlPcS_4_Cl dark cells. The results are depicted as the mean ± SEM of triplicate determinations from two independent experiments.

Several cellular stresses are activators of the intrinsic pathway of apoptosis. Cellular ROS have been observed to be associated with the initiation of the mitochondria-dependent cell death pathway. ROS can damage the mitochondria membrane, leading to the release of several mitochondrial proteins into the cytosol. These proteins interact with other proteins in the cytosol, resulting in the activation of caspases and, consequently, cell death (38). The DCFH-DA probe was used to determine the amount of ROS produced upon irradiation of 5µM AlPcS_4_Cl-PDT-treated HKESC-1 cells relative to the control cells. A significantly higher amount of ROS was seen in the cells exposed to AlPcS_4_Cl-PDT (*p*<0.001) (**Figure 9A**), demonstrating that ROS is a key driver of intrinsic cell death initiated by AlPcS_4_Cl-mediated PDT. Meanwhile, the laser-only and AlPcS_4_Cl presented similar ROS generations to those of the control cells.

Damage to the inner mitochondrial membrane potential (ΔΨm) is an outcome that is linked with the intrinsic pathway of apoptotic cell death (39). The effects of AlPcS_4_Cl on the mitochondrial integrity of oesophageal cancer cells were assessed using a rhodamine-123 efflux flow cytometry assay. Rhodamine-123 is a fluorescent dye that accumulates in intact mitochondria. An impaired mitochondria membrane results in reduced accumulation of the rhodamine dye, consequently leading to low fluorescence intensity. Rhodamine-123 efflux flow cytometry assay was employed to examine the mitochondria. In this assay, mitochondria with intact structures exhibit a high percentage of Δψm, and damaged mitochondria show a low percentage of Δψm. In **Figure 9B**, the percentage of Δψm was measured, and samples were compared in relation to the control cells. No statistical significance was observed among the control cell and AlPcS_4_Cl-only cells. In contrast, cells exposed to PDT at 5 J/cm^2^ demonstrated a statistically significant reduction in Δψm (***p< 0.001) compared to the control cells. Interestingly, laser-only (cells exposed to 5 J/cm^2^ irradiation with drug) showed a statistically significant decrease in Δψm (***p< 0.001).

#### 3.5.6 AlPcS_4_Cl-mediated PDT triggers cytochrome c release and caspase 3/7 Enzyme activity

**Figure 10:**
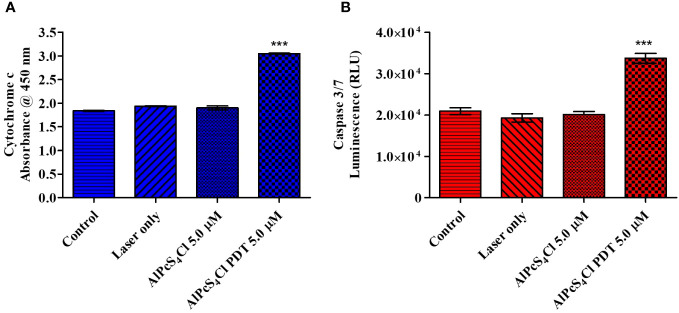
**(A)** Cytochrome c enzyme release mediated by AlPcS_4_Cl-PDT treated HKESC-1 oesophageal cancer cells. The AlPcS_4_Cl-PDT treated group exhibited a significant release of cytochrome c when examined in relation to the control, laser only, and AlPcS_4_Cl cells (***p<0.001). **(B)** Caspase 3/7 activity measurements on HKESC-1 oesophageal cancer cells exposed to AlPcS_4_Cl. The AlPcS_4_Cl-PDT treated group showed high significance of caspase 3/7 activity relative to the control, laser only, and AlPcS_4_Cl cells (***p<0.001). The bar graphs are shown as the mean ± SEM of three replicates from two individual experiments.

Cytochrome c plays a crucial role in oxidative phosphorylation and ATP generation. Damaged mitochondria trigger the release of several proteins, including cytochrome c. The levels of cytochrome c release were measured post-PDT. The results showed low cytochrome c levels in the control cells, laser-only, and AlPcS_4_Cl non-irradiated with no statistical difference observed (**Figure 10A**). The reduced cytochrome c levels demonstrated in the control cells, laser-only, and AlPcS_4_Cl non-irradiated cells implied undamaged and viable mitochondria. While the 5 µM AlPcS_4_Cl-PDT treated cells (**Figure 10A**) showed significantly high levels of cytochrome c (***p<0.001). This finding suggested impaired mitochondria, hence the efflux of cytochrome c protein into the cytosol. This outcome validates the intrinsic mitochondria apoptotic cell death pathway instigated by the AlPcS_4_Cl-mediated PDT on oesophageal cancer cells.

Caspases are proteolytic proteins required in the activation and enforcement of apoptosis. The initiation of these proteins is usually seen as a final indicator of apoptosis (40). Cytochrome c leakage from damaged mitochondria promotes cell death through the intrinsic apoptotic response by activating caspase 3/7 caspases, which are effector caspases (39). To further confirm the intrinsic apoptotic cell death induction by AlPcS_4_Cl-mediated PDT on oesophageal cancer cells, the caspase 3/7 luminescence assay was utilized to assess the activity of the caspase 3/7 enzyme. A significant increase in caspase 3/7 activity was observed, indicating a significant rise in the activity of this protein in the AlPcS_4_Cl-PDT treated group (***p<0.001) when compared with controls (**Figure 10B**).

#### 3.5.7 Autophagy evaluation of AlPcS_4_Cl-mediated PDT on oesophageal cancer

**Figure 11:**
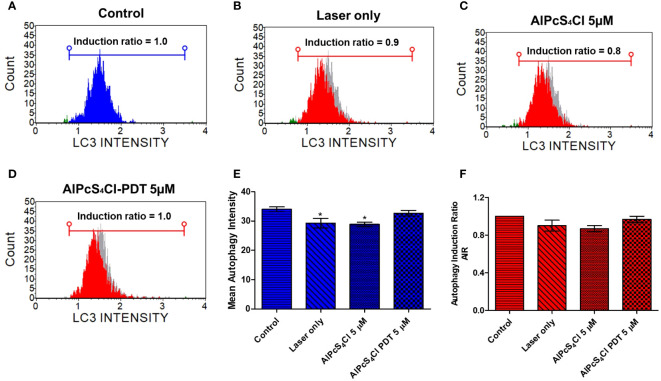
Autophagy cell death evaluation on oesophageal cancer cells. Histogram of mean autophagy intensity in the control and test groups **(A–D)**. **(E)** Percentage mean autophagy intensity of two separate autophagy triplicate assays, with the laser-only and AlPcS_4_Cl treated cell demonstrating a significant reduction in the mean autophagy intensity (*p<0.05). No significance was observed in the AlPcS_4_Cl-PDT-treated cells. **(F)** The autophagy induction ratio shows no significant induction in all the groups. The results are shown as the mean ± SEM of triplicate tests from two independent experiments.

The effects of AlPcS_4_Cl-mediated PDT on autophagy in oesophageal cancer cells were examined 24 hours after PDT using the Muse Autophagy LC3 antibody-based detection kit. The autophagy induction ratio was derived by using the ratio of the mean autophagy intensity of the test sample and the mean autophagy intensity of the control sample. Autophagy induction values ≥1 denote autophagy induction, and ≤ indicates no induction. The results in **Figures 11A–D** displayed the histogram of the mean autophagy intensity in the control and test groups. The control cells had an autophagy induction ratio of 1.0, depicting the absence of autophagy induction, laser-only treated cells 0.8, AlPcS4Cl treated cells without irradiation 0.9, and AlPcS_4_Cl-PDT treated cells 1.0. The percentage mean autophagy intensity of two separate autophagy triplicate assays in **Figure 11E** showed the laser-only and AlPcS_4_Cl treated cells had a significant reduction in the mean autophagy intensity (*p<0.05). No significant was observed in the AlPcS_4_Cl-PDT-treated cells. The autophagy induction ratio shows no significant induction in all the groups in **Figure 11F**.

## 4 Discussion

The application of AlPcS_4_Cl in PDT has been widely investigated on several cancers owing to its unique photochemical features (3, 20, 21, 23). The current study revealed that AlPcS_4_Cl-mediated PDT induced DNA damage response and intrinsic apoptosis pathway as observed by the localization of the PS in the mitochondria/nucleus, upregulation of ATM a DNA damage sensor, high ROS production, reduction in ATP level, decrease in ΔΨM, increase in apoptotic cells, and increment in cytochrome c and caspase 3/7 activity. First, we determine the spectral absorption feature of AlPcS_4_Cl to determine its absorption wavelength required for downstream PDT applications. One of the attributes of an effective PS is that it should possess a high absorption wavelength within the optical therapeutic window (600-800nm) for deeper tissue penetration (41). Our results showed that AlPcS_4_Cl display maximum light absorption (675nm wavelength) within the optical therapeutic window.

The intracellular localization of the PS, a parameter that determines the effectiveness of PDT, was examined. The site of accumulation of the PS dictates which intracellular organelles would be affected and the mode of cell death that would be activated (24). In this study, we observed the preferential sub-localization of AlPcS_4_Cl in mitochondria, lysosomes, ER and the nucleus. This implies that the AlPcS_4_Cl-mediated PDT mode of cell death could be dependent on these organelles. This agrees with a study by Ndhundhuma and colleagues demonstrating the localization of AlPcS_4_Cl in the mitochondria, lysosomes, and nuclei of melanoma cells (25). Also, other studies showed that AlPcS4Cl is accumulated in the mitochondria and lysosomes of lung tumor cells (20) and lung cancer stem cells (42). The internalization of the AlPcS_4_Cl in the nuclei of HKESC-1 oesophageal cancer cells suggests that this PS could induce cell death through the DNA damage response-dependent pathway.

Results from cell viability analysis using ATP cell viability assay demonstrated that the control cells without the drug and the cells administered with AlPcS_4_Cl (1.25- 20 µM) without irradiation (0 J/cm^2^) had active and viable cells with high ATP levels, indicating the non-toxicity action of AlPcS_4_Cl when inactive. However, AlPcS_4_Cl-mediated PDT exposed to 1.25 - 20 µM concentration irradiated at 5 J/cm^2^ significantly reduced cell viability and decreased ATP levels in a dose-dependent manner. The results also showed that AlPcS_4_Cl at 5 J/cm^2^ has an optimum phototoxic (IC_50_) effect of 5 µM on HKESC-1 oesophageal cancer cells. The cytotoxicity effects of AlPcS_4_Cl on HKESC-1 oesophageal cancer cells were assessed using the LDH release cytotoxicity assay, which detects the amount of enzymes relinquished into the culture medium following cell damage. The results showed a significantly higher LDH enzyme release in the AlPcS_4_Cl-mediated PDT group than in the control cells. This implies that AlPcS_4_Cl-mediated PDT promotes cytotoxicity in oesophageal cancer cells.

The mode of cell death induced by AlPcS_4_Cl-PDT in oesophageal cancer cells was carried out using various cell death assessment strategies. Findings from morphological assessment of cell death showed several morphological variations in the AlPcS_4_Cl-PDT group when compared to the control, which was suggestive of cell death. This finding validated the ATP cell viability and LDH cytotoxicity reports obtained in this study. Furthermore, this finding agrees with previous reports where they observed morphological distortion in cervical and lung cancer stem cells treated with zinc phthalocyanine and AlPcS_4_Cl-based PDT, respectively (42, 43). We evaluated the effect of AlPcS_4_Cl-PDT on the phases of the cell cycle in oesophageal cancer cells. The cell cycle is a well-coordinated multiple mechanism that regulates cell proliferation and DNA damage homeostasis to ensure that DNA lesions are not transferred to new daughter cells. The cell cycle is made up of four phases: gap 1(G1), synthesis (S), G2, and mitosis (M). Cell cycle checkpoints have emerged to regulate the cell cycle process in the event there is DNA damage (44). The proportion of cells in the G0/G1 phase significantly increased in the cancer cells exposed to AlPcS_4_Cl-PDT when compared to the control. Therefore, our findings imply that AlPcS_4_Cl-mediated PDT induced cell cycle arrest at the G0/G1 checkpoint. The precise mechanism by which AlPcS_4_Cl-mediated PDT interrupt cell cycle regulation requires further interrogation. Cell cycle mediating actions of AlPcS_4_Cl-PDT have been previously demonstrated to induce cell cycle arrest at the G0 phase in lung cancer (45). Likewise, zinc phthalocyanine tetra sulfonate-loaded nanoparticles were observed to inhibit the cell cycle at the G0 phase in colorectal cancer (46). Hence, the cell cycle arrest in the G0/G1 phase in HKESC-1 oesophageal cancer cells following exposure to AlPcS_4_Cl-mediated PDT may result from the accumulation of DNA damage.

One of the key causes of cell damage and death in PDT is oxidative DNA damage, which could be induced by free radicals. DNA damage may result in single or double-strand breaks (35, 47). When DNA damage occurs, repair mechanisms are activated to sustain the survival and stability of the DNA genome. This entire process in reaction to DNA damage is termed DNA damage response (DDR) (32). The effect of AlPcS_4_Cl-mediated PDT on DDR was examined. Exposure of HKESC-1 oesophageal cancer cells to AlPcS_4_Cl-mediated PDT significantly triggered double-strand break (DSB) DNA damage. It activated the upregulation of Ser1981 phosphorylated ATM, a DNA DSB sensor when compared to the control cells. A study by El-Hussein and colleagues demonstrated that zinc phthalocyanine-based PDT significantly induced DNA damage in lung, breast, and oesophageal cancer cells (36). Moreover, another study also showed that aluminium phthalocyanine tetra sulfonate (AlPcS_4_)-mediated PDT induced DNA damage in laryngeal cancer (35). The upregulation of ATM in response to DSB in our study is consistent with established evidence where ATM-mediated repair is believed to be the main mechanism in the DDR pathway to mend DSB lesions (33, 48). Furthermore, ATM functions in the regulation of cell cycle checkpoints following cellular stress, especially at the G0/G1, S, and G2/M cell cycle arrest (33, 49). This is in agreement with our cell cycle study, where we observed G0/G1phase cell cycle arrest. The activation of ATM results in the phosphorylation of several downstream proteins, such as H2A.X, which helps to intensify further the DNA DSB repair (34, 50). The downregulation of H2A.X in our study suggests that DNA repair may not have been successful. Cells exposed to DNA-damaging agents can decide to repair the DNA damage, tolerate the assaults or undergo cell death (32).

The mode of cell death mediated by AlPcS_4_Cl-based PDT on oesophageal cancer was performed using the annexin V-FITC/PI assay. The findings demonstrated that AlPcS_4_Cl-PDT trigger cell death via early apoptosis. However, a small percentage of necrosis was observed, which was lower than the control cells. No significant difference was seen between the AlPcS_4_Cl-PDT treatment group and the controls. This finding reported a similar cell death pattern observed by Ndhundhuma and Abrahamse (23) in melanoma cells treated with AlPcS_4_Cl mediated PDT using the Annexin V-FITC/PI assay. We also observed that the percentage of live cells in the control cells is low compared to the laser and AlPcS_4_Cl-only group. This observation is similar to the Annexin V-FITC/PI findings reported by Wei and colleagues in oesophageal cancer cells exposed to hematoporphyrin derivative (HpD)-PDT (51). The reason for this reduction is not clear.

Apoptosis has been observed to be activated by oxidative stress. We further examined if ROS initiated the observed induction of apoptosis. Apoptosis-linked ROS generation triggered by AlPcS_4_Cl-mediated PDT was investigated using the DCFDA/H2DCFD cellular ROS test. An increased amount of cellular ROS production was demonstrated in the cancer cells subjected to AlPcS_4_Cl-based PDT, establishing the activation of oxidative stress-related cellular damage in the oesophageal tumor cells. Increased ROS production may trigger DNA strand break, mitochondrial DNA impairment, and destruction of mitochondrial DNA (52). Oxidative stress and ROS have been implicated in promoting the mitochondria-dependent/intrinsic apoptotic cascade (53).

In normal cellular conditions, the mitochondria are the main source of cellular ATP production. However, in a disease state, the mitochondria serve as the primary source for oxygen radicals and ROS, blocking the normal function of ATP, preventing cell growth, facilitating the efflux of pro-apoptotic molecules and, consequently, cell death (38, 53). The effects of AlPcS_4_Cl on the ΔΨM of the HKESC-1 cancer cells were evaluated using the rhodamine-123 efflux flow cytometry assay. The findings demonstrated that AlPcS_4_Cl-upon irradiation significantly reduced the ΔΨM in the HKESC-1 oesophageal tumor cells. Also, this finding further confirmed the result from subcellular internalization, suggesting that the PS could trigger cell death via the intrinsic apoptotic pathway. This agrees with the finding that AlPcS_4_Cl reduce the ΔΨM in lung CSC (42). Reduction in ΔΨM is a critical step in the intrinsic apoptotic cascade. Impaired ΔΨM results in protein efflux from the inter-membrane space, such as cytochrome c, which activates other caspases involved in the activation and execution of apoptosis.

**Figure 12:**
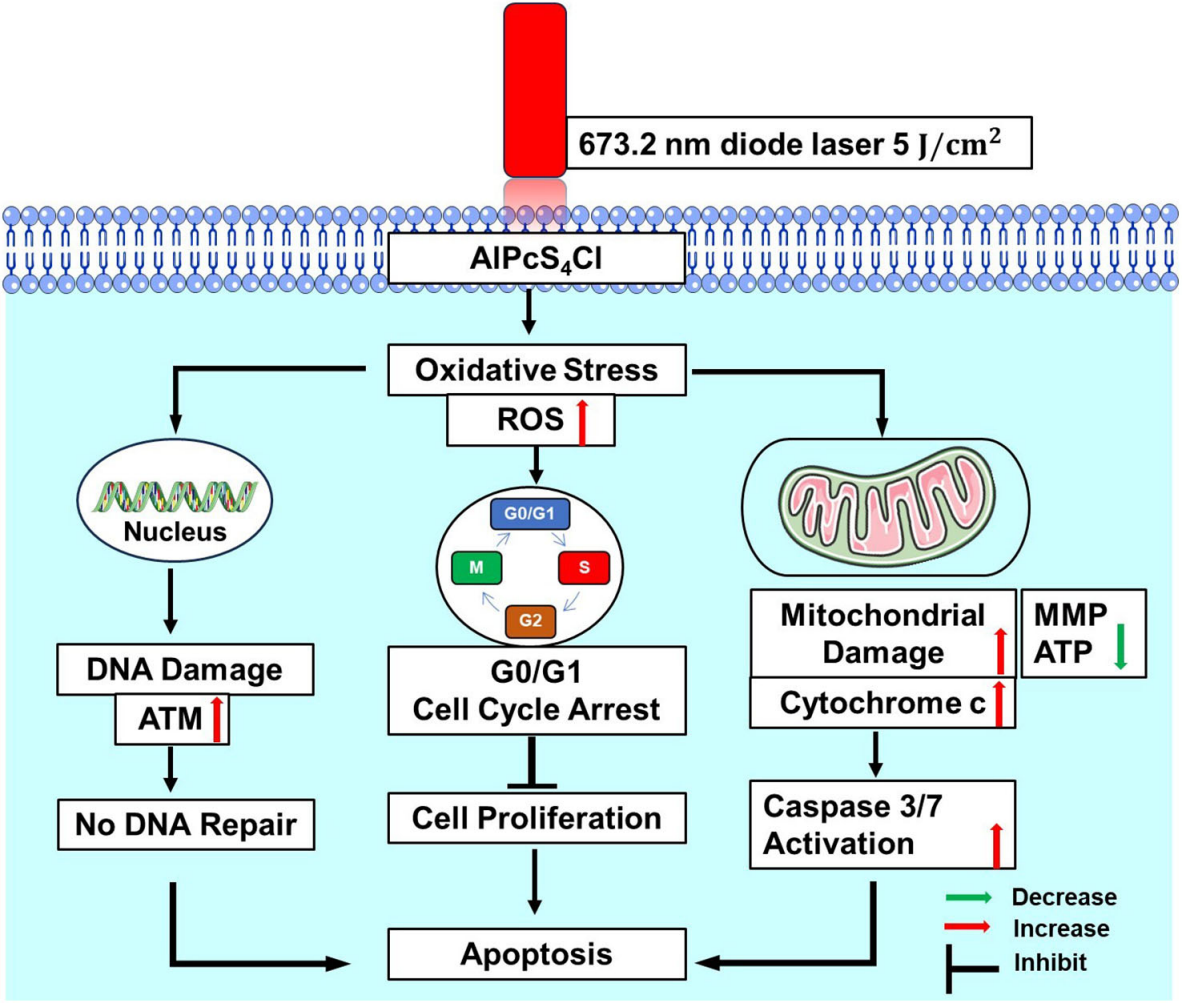
Proposed mechanism of AlPcS_4_Cl- mediated PDT triggered apoptosis in HKESC-1 oesophageal cancer cells through ROS generation, DNA damage, G0/G1 cell cycle arrest, mitochondrial damage, and caspase activation. ATM, ataxia-telangiectasia mutated; MMP, mitochondrial membrane potential; ATP, Adenosine triphosphate.

Cytochrome c is a protein involved in regulating cell growth and cell death. It functions in electron transfer as a component in the mitochondrial electron transport chain, and hence, it plays a vital role in the ATP generation process. It is also crucial in apoptosome formation and, consequently, induction of apoptosis (54). In this study, we employed ELISA to examine the levels of cytochrome c release in the cancer cells following treatment. High levels of cytochrome c release were demonstrated in the AlPcS_4_Cl-mediated PDT-treated cells than in the control cells. The findings validated that AlPcS_4_Cl-mediated PDT incurs damage to the mitochondria. This agrees with the study conducted by Mfouo-Tynga and colleagues (2018), where breast cancer cells treated with zinc-phthalocyanine-gold dendrimeric nanoparticles induced damage to mitochondria, resulting in the efflux of cytochrome c and apoptotic cell death (55). This also aligns with studies on human nasopharynx KB carcinoma cells (56) and melanoma cells (57) treated with zinc-phthalocyanine. The activation of the cytochrome c protein is an essential upstream process required to execute intrinsic apoptotic signaling pathways and caspase-dependent cell death. Caspase 3/7 activation is a crucial step that is required in the final execution of cell death in the apoptotic signaling pathway. In this study, the caspase 3/7 activity was elevated in the group exposed to AlPcS_4_Cl-mediated PDT. It has been established that a damaged ΔΨM releases various proteins that activate caspases, which are effectors of apoptosis (58). The high caspase 3/7 activity demonstrated here could be associated with the reduction in the ΔΨM observed in this study. Similar studies also reported activation of the caspase 3 enzyme in melanoma cells (57), human nasopharynx KB carcinoma cells, and breast cancer cells (55) exposed to zinc-phthalocyanine-mediated PDT. This further showed that AlPcS_4_Cl induce apoptotic cell death in oesophageal cancer via the intrinsic signaling cascade (**Figure 12**).

Finally, we examined the effects of AlPcS_4_Cl-mediated PDT on autophagic cell death using the Muse^®^ Autophagy LC3 antibody-based detection assay on oesophageal cancer cells. Autophagy induction in relation to the control oesophageal cancer cells was evaluated. Our findings revealed that oesophageal cancer cells exposed to AlPcS_4_Cl-mediated PDT had no or little effect on LC3-II formation, a marker of autophagosome formation, demonstrating no autophagy induction in relation to that of the control cells. Thus, AlPcS_4_Cl-mediated PDT triggered no autophagy cell death action in HKESC-1 oesophageal cancer cells. Conversely, a study with zinc-phthalocyanine-based PDT on melanoma cells was shown to induce autophagy (59).

## 5 Conclusions

The findings presented here demonstrate that AlPcS_4_Cl-mediated PDT is an effective anti-cancer agent as observed through the induction of oxidative stress, DNA damage response and apoptotic cell death in oesophageal cancer cells. This induction is displayed through increased ROS generation, DNA double-strand breaks via upregulation of ATM response, cell cycle arrest at the G0/G1 phase, cellular ATP reduction needed for mitochondria function, increased damage in the ΔΨM, efflux of cytochrome c and high caspase 3/7 activity, which is the final steps of the apoptosis cascade. Our study thus uncovers the DDR pathway and intrinsic apoptosis cell death mechanism of AlPcS_4_Cl-mediated PDT in oesophageal cancer cells. Therefore, the ultimate outcome of AlPcS_4_Cl-mediated PDT-treated cells relies on the interplay between DNA damage repair and pro-apoptotic cell death mechanisms stimulated in response to oxidative stress. The significant sensitivity of HKESC-1-treated oesophageal cancer cells to DNA damage and DDR presents an excellent approach for blocking the DNA repair mechanisms involved in oesophageal cancer cells. This approach can potentially improve the efficacy of AlPcS_4_Cl-mediated PDT. More studies on other cell lines are needed to validate these findings. Further studies are also required to identify the interaction between DNA damage response and apoptosis using inhibitors of the DNA repair pathway and AlPcS_4_Cl-mediated PDT.

## Data availability statement

The raw data supporting the conclusions of this article will be made available by the authors, without undue reservation.

## Ethics statement

Ethical approval was not required for the studies on humans in accordance with the local legislation and institutional requirements because only commercially available established cell lines were used.

## Author contributions

OCD: Conceptualization, Data curation, Formal analysis, Investigation, Methodology, Writing – original draft. RC: Supervision, Validation, Writing – review & editing. HA: Funding acquisition, Resources, Supervision, Writing – review & editing.

## Footnotes

1
https://www.graphpad.com/guides/prism/latest/curve-fitting/reg_dr_inhibit_normalized_variable.htm
